# SIRM-SIAAIC consensus, an Italian document on management of patients at risk of hypersensitivity reactions to contrast media

**DOI:** 10.1186/s12948-020-00128-3

**Published:** 2020-07-31

**Authors:** Maria Teresa Costantino, Laura Romanini, Francesco Gaeta, Fulvio Stacul, Rocco Luigi Valluzzi, Matteo Passamonti, Patrizia Bonadonna, Giovanni Cerri, Stefano Pucci, Paolo Ricci, Eleonora Savi, Michele Galluzzo, Marina Mauro, Emanuele Grassedonio, Mona Rita Yacoub, Alfonso Reginelli, Sergio Testi, Erminia Ridolo, Eustacchio Nettis, Elisabetta Di Leo, Oliviero Rossi, Paolo Montuschi, Cristoforo Incorvaia, Antonino Romano

**Affiliations:** 1 Allergy Unit, Department of Medicine, ASST Mantova, Mantua, Italy; 2 Radiology, Istituti Ospitalieri di Cremona, ASST Cremona, Cremona, Italy; 3 Allergy Unit, Columbus Hospital, Fondazione Policlinico Universitario Agostino Gemelli IRCCS, Via Moscati n.30, Rome, Italy; 4 Department of Radiology, Ospedale Maggiore, Azienda Sanitaria Universitaria Integrata di Trieste, Trieste, Italy; 5 Allergy Unit, Bambino Gesù Children’s Hospital, IRCCS, Rome, Vatican City, Italy; 6 Department of Radiology-ASST di Lodi, Lodi, Italy; 7 Allergy Unit, Azienda Ospedaliera Universitaria Integrata of Verona, Verona, Italy; 8 Department of Radiology, ASST Spedali Civili di Brescia, Brescia, Italy; 9 Allergy Unit. General Hospital, Civitanova Marche, Milan, Italy; 10 Department of Radiology, Oncologiche ad Anatomopatologiche, Azienda Policlinico Umberto I, Sapienza Università di Roma, Rome, Italy; 11 Departmental Unit of Allergology, Guglielmo da Saliceto Hospital, Piacenza, Italy; 12 Department of Radiology, Azienda Ospedaliera San Camillo Forlanini, Ospedale San Camillo, Rome, Italy; 13 Allergy Unit, S. Anna Hospital, Como, Italy; 14 Department of Radiology, Dipartimento di Biopatologia e Biotecnologie Mediche, Policlinico Paolo Giaccone, Università degli Studi di Palermo, Palermo, Italy; 15 Allergy and Immunology Department, IRCCS San Raffaele Hospital, Milan, Italy; 16 Department of Radiology & Radiotherapy, University of Campania ‘Luigi Vanvitelli’, Naples, Italy; 17 Allergy and Clinical Immunology Unit, San Giovanni di Dio’s Hospital, Florence, Italy; 18 Department of Medicine and Surgery Clinical, University of Parma, Via Gramsci 14, 43126 Parma, Italy; 19 Department of Emergency and Organ Transplantation, School of Allergology and Clinical Immunology, University of Bari Aldo Moro, Bari, Italy; 20 Section of Allergy and Clinical Immunology, Unit of Internal Medicine, “F. Miulli” Hospital, Acquaviva delle Fonti, Bari, Italy; 21 Allergy Unit, Azienda Ospedaliero Universitaria Careggi, Florence, Italy; 22 Pharmacology Unit, Fondazione Policlinico Universitario Agostino Gemelli IRCCS, Rome, Italy; 23 Department of Pharmacology, Faculty of Medicine, Catholic University of the Sacred Heart, Rome, Italy; 24 Cardiac/Pulmonary Rehabilitation, ASST Pini-CTO, Milan, Italy; 25 IRCCS Oasi Maria S.S., Troina & Fondazione Mediterranea G.B. Morgagni, Catania, Italy

**Keywords:** Radiologic contrast media, Hypersensitivity reactions, Low-osmolar contrast agents, Diagnosis, Management

## Abstract

Hypersensitivity reactions (HRs) to contrast media (CM) can be distinguished in immune-mediated (including allergic reactions) and non-immune-mediated reactions, even if clinical manifestations could be similar. Such manifestations range from mild skin eruptions to severe anaphylaxis, making it important for radiologists to know how to identify and manage them. A panel of experts from the Società Italiana di Radiologia Medica e Interventistica (SIRM) and the Società Italiana di Allergologia, Asma e Immunologia Clinica (SIAAIC) provided a consensus document on the management of patients who must undergo radiological investigations with CM. Consensus topics included: the risk stratification of patients, the identification of the culprit CM and of a safe alternative by an allergy workup, as well as the use of premedication and the correct procedure to safely perform an elective (i.e., scheduled) or urgent examination. The most important recommendations are: (1) in all patients, a thorough medical history must be taken by the prescribing physician and/or the radiologist to identify at-risk patients; (2) in patients with hypersensitivity reactions to CM, the radiologist must consider an alternative, non-contrast imaging study with a comparable diagnostic value, or prescribe a different investigation with another class of CM; (3) if such options are not feasible, the radiologist must address at-risk patients to a reference centre for an allergy evaluation; (4) if timely referral to an allergist is not viable, it is recommended to use a CM other than the responsible one, taking into account cross-reactivity patterns; in the case of patients with histories of severe reactions, the presence of an anesthesiologist is also recommended and a premedication is suggested.

## Background

Adverse reactions to contrast media (CM) are a relevant problem due to the tremendous increase of CM administration for diagnostic and therapeutic procedures. Two types of adverse reactions to CM can be distinguished: (a) toxic reactions, which are considered to be predictable, dose-dependent, and related to chemical properties of CM; (b) hypersensitivity reactions (HRs), which, based on timing of symptom appearance, are classified as immediate (< 1 h) and nonimmediate (also called delayed, > 1 h after CM administration) [1, 2]. Immediate HRs (IHRs) have been reported in 0.7% to 3% of patients receiving nonionic CM, severe reactions in 0.02% to 0.04% of intravenous procedures, and fatal IHRs in 0.00001% to 0.0003% of ICM applications [3]. Two studies demonstrated a 0.6% and 0.2% rate of allergy-type reactions, and 0.01% and 0.005% of the total number of nonionic ICM injections were classified as severe reactions [4, 5]. Recently, Lee et al. [6] reported that during the period of their study the overall incidence of IHRs to iodinated CM (ICM) was 1% and the incidence of severe reaction was 0.02%. Regarding gadolinium-based contrast media (GBCM), the rate of hypersensitivity reactions to them (mostly immediate) ranges from 0.07 [7] to 0.3% [8], whereas the rate of severe IHRs ranges from 0.003 [5, 9, 10] to 0.008% [11], with a death rate of less than 1 in a million [9]. Urticarial eruptions are the common clinical symptoms of HRs to GBCM [12].

Although rare (1/50,000 to 1/200,000), severe reactions to CM require early recognition and awareness of the radiological team [13]. Significantly, the rarity of severe life-threatening reactions to CM is the reason because good quality of evidence data on their diagnosis and prevention are lacking and, therefore, the present document will not be largely evidence based. Exanthematic nonimmediate hypersensitivity reactions (NIHRs) affect 0.5% to 3% of ICM-exposed patients [2]. A higher incidence of nonimmediate exanthemas associated with dimeric nonionic CM has been reported [14].

With regard to pathogenic mechanisms, IHRs have been considered for decades to be non-allergic, resulting from non-specific activation of basophils and other biochemical mechanisms, such as the effect of CM hyperosmolarity or complement activation [1, 2]. Over the last 20 years, cumulative evidence has been published in the literature about the involvement of an IgE-mediated pathogenic mechanism in some IHRs to ICM and GBCM [1, 2, 13]. Regarding NIHRs, some ICM-induced skin eruptions, especially maculopapular exanthemas, appear to be associated with a T cell-mediated pathogenic mechanism [1, 2, 14]. Maculopapular exanthemas due to a T-cell-mediated hypersensitivity to GBCM have rarely been reported [15].

**Fig. 1:**
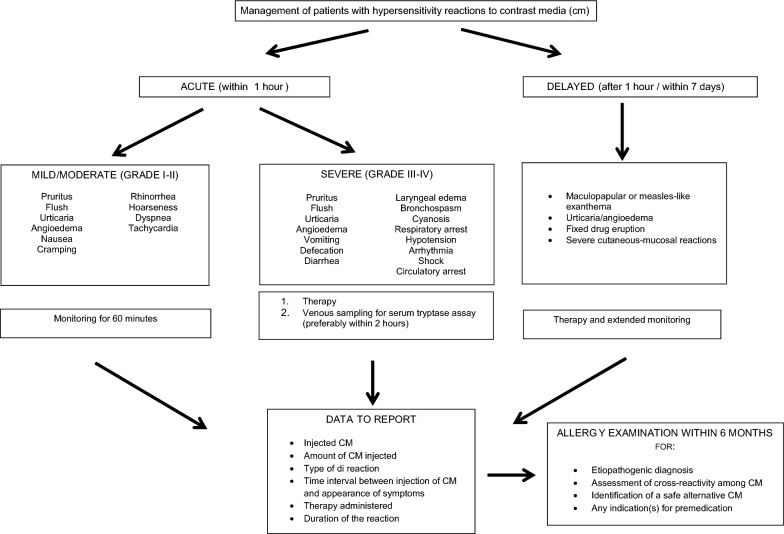
Management of patients with hypersensitivity reactions to contrast media (cm)

The clinical manifestations of HRs are shown in Fig. 1. Considering the CM large use, it is important for radiologists to identify and manage HR clinical pictures ranging from mild skin eruptions to severe anaphylaxis. For grading IHRs, the severity scale of Ring and Messmer [16] can be used (Table 1). According to Brockow et al. [17], NIHRs can be classified as mild when no treatment was required, moderate when the patient responded readily to appropriate treatment and no hospitalization was needed, and severe when the reaction required hospitalization or was life-threatening.

**Table 1:** Grading of the severity of immediate hypersensitivity reactions to CM [16]

Symptoms					
Grade	Skin		Abdomen	Respiratory tract	Circulation
I	Pruritus Flush Urticaria Angioedema				
II	Pruritus Flush Urticaria Angioedema	(Not obligatory)	Nausea Cramping	Rhinorrhea Hoarseness Dyspnea	Tachycardia (δ > 20/min)
III	Pruritus Flush Urticaria Angioedema	(Not obligatory)	Vomiting Defecation Diarrhea	Laryngeal edema Bronchospasm Cyanosis	Hypotension (δ > 20 mmHg syst.) Arrhythmia Shock
IV	Pruritus Flush Urticaria Angioedema	(Not obligatory)	Vomiting Defecation Diarrhea	Respiratory arrest	Circulatory arrest

A panel of radiologists belonging to the Società Italiana di Radiologia Medica e Interventistica (SIRM) and allergists belonging to the Società Italiana di Allergologia, Asma e Immunologia Clinica (SIAAIC), expert of hypersensitivity reactions to CM, met in Florence on April 2017 (during the 30th SIAAC Congress) and in Mantua on July 2017, with the aim of drawing up a consensus document on the management of patients who must undergo radiological investigations with CM. Consensus topics included: the risk stratification of patients—based on the history of any hypersensitivity reactions to CM and current information on the patient’s clinical condition (e.g., cardiopulmonary status, any concomitant diseases etc.) and active medications (e.g., beta-blockers, interleukin-2)—, the identification of the culprit CM and of a safe alternative, as well as the use of premedication and the correct procedure to safely perform an elective or urgent examination. The document took into account the existing European guidelines [1, 18, 19] and is intended for physicians and health professionals. Where the literature was poor, the collective experience of members of the expert panel was taken into consideration.

## Risk stratification of patients

Prior to an imaging study with CM, a thorough medical history must be taken by the prescribing physician and/or the radiologist to identify at-risk patients. Table 2 shows the risk stratification of patients in 3 groups: group 2 (high-risk patients), with histories of hypersensitivity reactions (mild, moderate, or severe) to CM of the same chemical class of the one to be administered (i.e., GBCM or ICM) [1, 7, 17, 18, 20]; group 1 (low-risk patients), with concomitant diseases, such as uncontrolled asthma [21, 22], active urticaria-angioedema [2, 22], mastocytosis [19]; and group 0 (subjects at risk very low), for example those with histories of allergic reactions to causative agents other than CM, such as foods [23], drugs [24], and iodine-containing antiseptics (e.g., iodopovidone or iodoform) [25].

**Table 2:** Risk stratification according to the patients’ characteristics

Group	Risk factors	Level of risk	Allergy evaluation for CM
0	Female gender [19] Atopy [19] Intravascular CM injection [23] Severe cardiovascular disease [23] Viral infection and autoimmune diseases [1] Psychiatric disorders [23] Interleukin-2 treatment and contact allergy (for NIHRs) [2] Treatment with ACE-inhibitors, beta-blockers, or proton pump inhibitors (for IHRS) [19] Food allergy [23] Drug allergy [24] Concomitant allergic disease [23] Allergy to iodine-containing antiseptics [25]	Very low (not relevant)	No
1	Uncontrolled asthma [21, 22] Active urticaria-angioedema [2, 22] Mastocytosis [19] Recurrent angioedema Idiopathic anaphylaxis	Medium	No
2	History of hypersensitivity reactions to contrast media of the same class of the one to be administered [1, 7, 17, 18, 20]	High	Yes

## Premedication

With regard to premedication, allergists and radiologists generally differ in the approach, and consensus multidisciplinary strategies (and even care pathways) should be established to overcome differences between specialists [26]. Overall, in adult and pediatric patients at high risk for allergic reactions to CM, data on the effectiveness of premedication with glucocorticoids associated or not with antihistamines are not univocal [27–33]. Especially for group 2 patients with a history of severe reactions to CM, the optimal approach has not yet been established, mainly because of the rarity of such reactions. There is no standardized premedication regimen, with differences between the North American and European recommendations. Specifically, premedication is not recommended by ESUR guidelines on CM [18] because “there is not good evidence of its effectiveness”. Instead, premedication with glucocorticoids, antihistamines, and sympathomimetics to prevent severe reactions to CM is the standard of care in all US institutions [26, 34]. Anyway, a systematic review by Tramèr et al. [30], referring to a study by Lasser et al. [28], showed a good quality of evidence for the effectiveness of premedication with methylprednisolone 32 mg administered orally 12 and 2 h before CM injection on severe (potentially life-threatening) and mild reactions. In another study [6], among 850 patients with histories of IHRs to ICM who underwent enhanced computed tomography after severity tailored prophylaxis according to the American College of Radiology manual on CM [34], only 16 patients (1.9%) experienced a recurrence of moderate to severe HRs. In this study [6], the premedication regimen for patients with a severe index reaction consisted of intravenous administration of 40 mg of methylprednisolone 4 h and 1 h before the ICM injection and of 4 mg of chlorpheniramine 1 h before the examination. In addition, an ICM other than the one responsible was used. Recently, an expert group of HRs to CM considered that premedication can be reserved to decrease reaction frequency or severity in high-risk patients, including those who experienced severe IHRs and present negative results in the allergy workup [26]. In patients who reported severe NIHRs (e.g., DRESS, SJS/TEN, etc.), premedication is contraindicated, as well as the suspected CM, and a non-cross-reactive alternative ICM will need a careful evaluation [26].

In the present consensus, we agreed almost completely with the ESUR guidelines [18] that recently removed the suggestion of using premedication in patients at risk. In fact, we suggested premedication regimens only in group 2 patients with severe reactions to unknown CM and in group 1 patients with recurrent angioedema, mastocytosis, or idiopathic anaphylaxis, both in elective and urgent examinations. Prospective randomized controlled clinical trials are needed to develop premedication regimens of proven effectiveness. Therefore, the premedication schemes of this consensus article must be considered temporary and their use not mandatory.

## Management of patients at risk

**Fig. 2:**
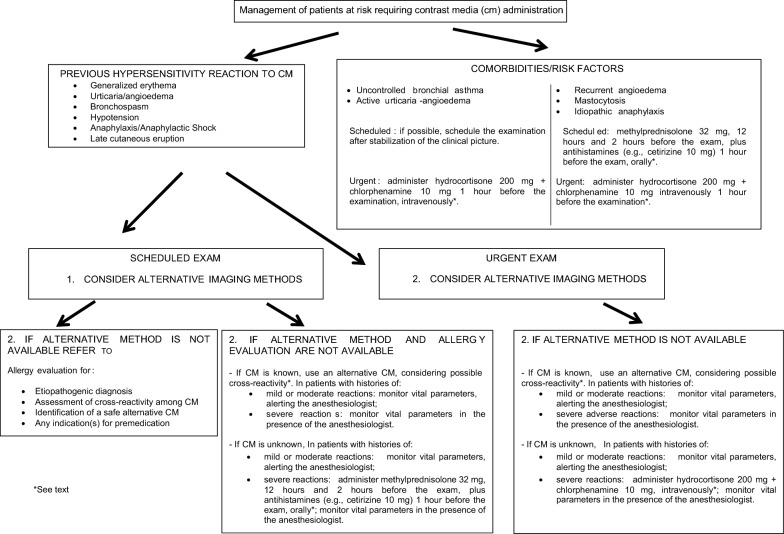
Management of patients at risk requiring contrast media (cm) administration

Imaging studies with CM generally fall into one of two categories: “elective examinations” (usually outpatient) and “urgent examinations” (Fig. 2).

### Elective examination

In patients with histories of hypersensitivity reactions to CM, the radiologist must consider an alternative, non-contrast imaging study with comparable diagnostic value, or prescribe a different examination with other classes of CM (i.e., GBCM or microbubbles in the case of reaction to ICM, or vice versa). If an alternative imaging examination is not available or not useful for the pathology to be studied, the radiologist must address patients to a reference centre for an allergy evaluation. The allergist will propose an appropriate diagnostic procedure according to European guidelines [1, 18, 19]. The radiologist can also refer patients with bronchial asthma, active urticaria-angioedema, recurrent angioedema, mastocytosis, or idiopathic anaphylaxis to the allergist, especially if they have not been checked for a long time. The allergist should provide a treatment to stabilize the patient’s condition in the case of uncontrolled asthma and/or active urticaria-angioedema (Fig. 2). Based on the characteristics of the index reaction (e.g., mild, moderate, or severe) and the patient’s risk profile, the allergist may propose a premedication.

When timely referral to an allergist it is not feasible (Fig. 2):

- In patients with histories of mild, moderate, or severe index reaction (Table 1) to known CM, on the basis of the literature data [18, 19, 22, 32], we recommend to use a different CM, albeit of the same class, taking into account the cross-reactivity patterns among CM (see below); in case of a severe index reaction (see Appendix A), it is important to monitor vital parameters during the procedure in the presence of the anesthesiologist [18]. Significantly, in a study by Park et al. [22] concerning patients with prior moderate-to-severe HRs to low-osmolar ICM, the risk of recurrent HRs was 67.1% lower in cases where the implicated ICM was changed to another one (odds ratio: 0.329; P = 0.001), whereas glucocorticoid premedication did not show protective effects against recurrent HRs.
- In patients with histories of mild or moderate index reactions (Fig. 1) to unknown CM, we suggest to monitor vital parameters, alerting the anesthesiologist; in group 2 patients with severe index reactions to unknown CM, taking into account the results of two studies [6, 28], we suggest to administer methylprednisolone 32 mg, 12 h and 2 h before the examination, plus antihistamines (e.g., cetirizine 10 mg) 1 h before the examination, orally. Moreover, we recommend to monitor vital parameters in the presence of the anesthesiologist.
- In group 1 patients with recurrent angioedema, mastocytosis, or idiopathic anaphylaxis, we suggest the above premedication regimen.
- In group 1 patients with active urticaria or uncontrolled bronchial asthma, we recommend to postpone the examination until the clinical symptoms stabilize. Alternatively, the premedication protocol suggested for urgent examinations (see below) can be considered.

### Urgent examination

Before an emergency examination with CM, the radiologist must collect a thorough medical history to identify at-risk patients, especially those belonging to group 2. In such patients, the radiologist should consider an alternative, non-contrast radiological examination with the same diagnostic effectiveness, or prescribe a different class of CM. If an alternative examination is not available:

- In patients with histories of hypersensitivity reactions (mild, moderate, or severe) to known CM, we recommend to use a different CM of the same class, taking into account cross-reactivity patterns (see below); in case of severe index reactions (see Appendix A), it is important to monitor vital parameters during the procedure in the presence of the anesthesiologist.
- In patients with histories of mild or moderate index reactions (Fig. 1) to unknown CM, we suggest to monitor vital parameters, alerting the anesthesiologist.
- In group 2 patients with severe index reactions, taking into account the results of a study by Greenberger et al. [27], we recommend to administer hydrocortisone 200 mg plus chlorphenamine 10 mg, intravenously, 1 h before the examination, monitoring vital parameters during the procedure in the presence of the anesthesiologist.
- In group 1 patients, we suggest the above premedication scheme and to monitor vital parameters.

In the setting of emergency examination with CM, it is important to note that, since the primary mechanism of action of glucocorticoids is modulation of gene expression, their therapeutic effect begins no earlier than 30 min after administration (even intravenous one) [35]. Note that there has been some concern related to adverse effects induced by systemic glucocorticoids, even when taken for short periods of time [33, 36]. In any case, physicians routinely involved in diagnostic procedures with CM should not rely on the effectiveness of premedication and should be trained in order to have specific skills in the diagnosis and treatment of anaphylactic reactions [26].

### The role of the anesthesiologist

In patients with histories of severe reactions (see Appendix A) to unknown CM, in both elective and urgent examinations, we advise that radiological investigations be performed in a safe environment and in the presence of the anesthesiologist, due to the undemonstrated effectiveness of premedication [29, 31–33] and the risk of cross-reactivity among CM of the same class, both iodine-based [13, 17, 37–48] and gadolinium-based [49].

## Management of patients with a hypersensitivity reaction to CM (Group 2)

In moderate or severe IHRs (Fig. 1), serum tryptase assay should be performed within 2 h and up to 24 h from the reaction, whenever possible. A twofold increase over the baseline value is suggestive of anaphylaxis [50]. Detection of possible blood eosinophilia is recommended in subjects with delayed reactions [1, 19]. For an appropriate allergy evaluation, the radiologist must report the adverse reaction, specifying the CM concerned, the dose administered, the time interval between the administration of CM and the appearance of symptoms, the characteristics of the adverse manifestation, the therapy administered, and the duration of the reaction.

### Allergy testing

#### Skin tests

Allergy testing is recommended within the 6 weeks to 6 months following the HR [13, 17]. According to a position paper regarding an International Consensus on drug allergy [51], the clinical tools allowing a definitive diagnosis include a thorough clinical history, standardized skin tests, and drug provocation tests (DPTs). The initial evaluation for IHRs includes skin testing with responsible CM. In case of positive responses or if the culprit CM is unknown, a broad panel of CM is tested [13, 45, 46]. Patient with histories NIHRs are evaluated by prick and intradermal tests with late readings, as well as patch tests [1, 17]. Positive skin testing confirms the allergic pathogenesis and gives sufficient evidence to contraindicate the culprit CM together with the other agents inducing skin cross-reactivity for the rest of the patient’s life [13, 17]. A meta-analysis of studies on skin tests in patients with HRs to ICM showed that these tests may be helpful in diagnosing and managing such patients, especially those with severe IHRs [52]. A subsequent prospective multicenter study evaluated 245 subjects with IHRs to ICM or GBCM by performing skin tests [13]. All 10 iodinated and 5 gadolinium agents on the French market were tested. Reactions were classified as allergic when intradermal tests were positive to the culprit CM solution diluted to the tenth, as recommended by the European Network for Drug Allergy [53], potentially allergic when skin tests were positive only with the pure solution, and non-allergic otherwise. Forty-one (19.6%) of the 209 IHRs to ICM and 10 (27.8%) of the 36 to GBCM were classified as allergic. The frequency of allergy increased with the severity of the reaction (9.5% in cutaneous reactions; 22.9% in moderate systemic ones, 52.9% in life-threatening ones, and 100% in cardiac arrest). Skin cross-reactivity with non-culprit CM diluted to the tenth was found in 31.4% of the allergic patients, and in 62.7% of them with pure solutions. Forty-two patients with well-defined IHRs had negative intradermal tests with diluted solutions but positive ones with pure solutions of CM, 34 with ICM and 8 with GBCM. Taking into consideration the results of this study [13] and those of a study by Torres et al. [40], which regarded subjects with NIHRs to ICM, it seems advisable to perform intradermal tests with CM up to the pure solution in order to increase both the sensitivity of diagnosis and the detection of cross reactivity. Recently, members of an expert panel of ICM allergy reiterated the importance of skin tests in the management of patients with HRs to ICM. According to them, the evaluation of patients with ICM-induced anaphylaxis or exanthema should always include appropriate skin tests ensuring that patients with IgE-mediated or delayed-type allergy are not missed [26]. Moreover, skin testing with a large panel of CM may identify alternative CM that could be tolerated in future radiologic investigations [13, 38–48].

#### Drug provocation test

The diagnosis of HRs to CM is challenging when based on clinical history and skin tests due to the limited sensitivity of these tools. According to the aforementioned document [51], the DPT, or challenge, is the gold standard for the identification of the drug eliciting a HR. Considering that the DPT is not indicated when the offending drug is unlikely to be needed [51] and that the DPT with CM involves the risk of severe reactions, there is a general consensus in recommending that it be carried out only in selected cases using a skin-test negative CM to identify an alternative compound for future use [19, 26, 46]. Interestingly, in studies that performed DPTs with negatively skin-tested alternative ICM in at least 10 subjects [39, 40, 44–48], the rate of positive responses to DPTs ranged from 0 [39, 46, 48] to 36.1% (13 of 36) [40].

In some studies [37, 40, 42, 43, 45, 47, 54], DPTs with suspected ICM were performed to verify negative in vivo and in vitro test results. DPT protocols varied widely among studies in terms of dose steps, time intervals between incremental doses, days of dosing, and maximum administered dose (i.e., from 10 to 120 mL). In studies that specified the number of DPTs performed with the suspected ICM found negative in skin testing [37, 40, 42, 45, 47, 54], the rate of positive responses ranged from 3.1 [54] to 34.6% [40]. In any case, according to the aforementioned panel of expert [26], the DPT with the suspected ICM is not recommended because can put patients at risk for a reaction outside a controlled environment and no controlled studies have provided evidence of its usefulness. The aforementioned meta-analysis demonstrated a negative predictive value (NPV) of skin tests for IHRs (n = 116) and NIHRs (n = 209) of 94.8% and 68.4%, respectively [52]. Altogether, the NPV seems higher for patients with an IHR compared with a NIHR. The NPV of GBCM skin testing is also interesting. In a study [55], 11 patients with HRs to GBCM were re-exposed to negatively skin-tested GBCM and tolerated them.

SIAAIC and SIRM discourage skin testing with CM as a pre-examination screening test in patients not presenting previous reactions to CM [56, 57], including those with atopy and/or drug allergy.

#### Cross-reactivity

Cross-reactivity among ICM has been reported, especially in subjects with delayed reactions [13, 17, 37–48] and seems to depend, at least in part, on the chemical structure of the CM [39, 43, 45]. The ICM most frequently involved in cross-reactions are iodixanol, iohexol, iopentol, ioversol, and iomeprol. In particular, the cross-reactivity between iodixanol and its monomer iohexol [17, 38–40, 42] should be noted. In a retrospective study by Lerondeau et al. [43], cross-reactions among ICM did not seem to be related to their chemical classification. The authors identified 3 groups of ICM by multiple correspondence analyses: group A (iodixanol, iopamidol, iomeprol, iohexol, ioversol, and ioxitalamate); group B (ioxaglate and iobitridol) the members of which share 2 identical *N*-(2,3-dihydroxypropyl) carbamoyl side chains (except ioxitalamate); and group C (amidotrizoate). This classification was based primarily on the scarcity of cross-reactivity among these 3 groups and the frequency of cross-reactivity among ICM within groups A and B. However, this study involved 97 patients, with only 2 anaphylactic shocks and therefore the quality of the evidence of the data concerning IHRs is very low. Recently, a study by Schrijvers et al. [45] evaluated 597 patients with HRs to ICM (423 immediate, 118 nonimmediate, and 56 with undetermined chronology) by performing skin tests with a panel comprising up to 10 ICM. Eighty patients (13.4%) were skin-test positive. Of the 54 patients with skin-test positivity on immediate reading, 37 had only 1 positive skin test and 17 had a cross-reactivity (range 2–5 of 10 tested ICM). Of the 26 subjects with skin-test positivity on delayed reading, 8 had only a single positive test, whereas 18 presented a cross-reactivity (range 2–10 of 10 tested ICM). Based on these results, the authors proposed a classification of ICM in three groups: group A, which included iodixanol, iomeprol, ioversol, iohexol, and iopromide that share an *N*-(2,3-dihydroxypropyl) carbamoyl side chain; group B which consisted of iobitridol, sodium ioxaglate, ioxitalamate, and iopamidol; and group C, with sodium amidotrizoate. Cross-reactivity was most frequently observed between iopromide and iomeprol (41.1%) for immediate and between ioversol and iomeprol (55.5%) for nonimmediate test positivity and mostly within the group A. Two hundred and thirty-three (39.0%) of the 597 subjects were re-exposed at least once to negatively skin-tested ICM: 217 (93.1%) of 233 patients tolerated them and 16 (6.9%) reacted. Of these 16 patients, 10 had a history of an IHR and 6 of an NIHR. Taking into account the results of this study [45], in subjects allergic to ICM it is recommended to perform skin tests with a large panel of alternative ICM and choose the alternative skin-test negative ICM from those belonging to a group other than that of the responsible ICM.

Cross-reactivity among gadolinium chelates is still unclear. In any case, a recent study demonstrated that this cross-reactivity mostly concerns gadoteric acid and gadobutrol [49].

## Conclusions

The literature data strongly support a reorganization of radiology departments, with better identification of previous reactors, elimination of systematic premedication, and a structured collaboration between radiologists and drug allergists to whom patients who experienced HRs to CM should be addressed possibly within 6 weeks to 6 months following the reaction [13, 17]. In the last decade, indeed, several studies demonstrated the usefulness of performing skin testing with a large panel of CM in patients with a clinical reaction resembling allergy and using a negatively skin tested CM afterward, without a systematic use of premedication [13, 17, 40, 43, 45–48]. In selected cases, challenges can be carried out only using skin-test negative CM in order to identify alternative compounds for future use.

## Data Availability

Literature data are listed in the References section.

## Appendix A

### Clinical criteria for diagnosing anaphylaxis^(a–c)^

1. Acute onset of an illness (minutes to several hours) with involvement of the skin, mucosal tissue, or both (e.g., generalized hives, pruritus or flushing, swollen lips-tongue-uvula) and at least one of the following:
  - Respiratory compromise (e.g., dyspnea, wheeze-bronchospasm, stridor, reduced PEF, hypoxemia).
  - Reduced BP or associated symptoms of end-organ dysfunction (e.g., hypotonia [collapse], syncope, incontinence).
2. Two or more of the following occur rapidly after exposure to a likely allergen for that patient (minutes to several hours):
  - Involvement of the skin-mucosal tissue (e.g., generalized hives, itch-flush, swollen lips-tongue-uvula).
  - Respiratory compromise (e.g., dyspnea, wheeze-bronchospasm, stridor, reduced PEF, hypoxemia).
  - Reduced BP or associated symptoms (e.g., hypotonia [collapse], syncope, incontinence).
  - Persistent gastrointestinal symptoms (e.g., crampy abdominal pain, vomiting).
3. Reduced BP after exposure to known allergen for that patient (minutes to several hours):
  - Infants and children: low systolic BP (age specific) or greater than 30% decrease in systolic BP*.
  - Adults: systolic BP of less than 90 mm Hg or greater than 30% decrease from that person’s baseline.

*PEF*, peak expiratory flow; *BP*, blood pressure.

**Low systolic blood pressure for children is defined as less than 70* *mm Hg from 1* *month to 1* *year, less than (70* *mm Hg *+* [2 × age]) from 1 to 10* *years, and less than 90* *mm Hg from 11 to 17* *years.*

- FE Simons, LR Ardusso, MB Bilò, et al. World Allergy Organization anaphylaxis guidelines: summary. J Allergy Clin Immunol 2011;127:587-93.e1-22.
- A Muraro, G Roberts, M Worm, et al. Anaphylaxis: guidelines from the European Academy of Allergy and Clinical Immunology. Allergy 2014;69:1026-45.
- HA Sampson, A Muñoz-Furlong, RL Campbell, et al. Second symposium on the definition and management of anaphylaxis: summary report-Second National Institute of Allergy and Infectious Disease/Food Allergy and Anaphylaxis Network symposium. J Allergy Clin Immunol 2006;117:391-7.

## References

[1] Brockow K, Christiansen C, Kanny G, Clément O, Barbaud A, Bircher A, et al. Management of hypersensitivity reactions to iodinated contrast media. Allergy. 2005;60:150–8. 15647034 10.1111/j.1398-9995.2005.00745.x

[2] Brockow K, Sánchez-Borges M. Hypersensitivity to contrast media and dyes. Immunol Allergy Clin North Am. 2014;34:547. 25017677 10.1016/j.iac.2014.04.002

[3] Katayama H, Yamaguchi K, Kozuka T, Takashima T, Seez P, Matsuura K. Adverse reactions to ionic and nonionic contrast media. A report from the Japanese Committee on the Safety of Contrast Media. Radiology. 1990;175:621–8. 2343107 10.1148/radiology.175.3.2343107

[4] Wang CL, Cohan RH, Ellis JH, Caoili EM, Wang G, Francis IR. Frequency, outcome, and appropriateness of treatment of nonionic iodinated contrast media reactions. AJR Am J Roentgenol. 2008;191:409–15. 18647910 10.2214/AJR.07.3421

[5] Hunt CH, Hartman RP, Hesley GK. Frequency and severity of adverse effects of iodinated and gadolinium contrast materials: retrospective review of 456,930 doses. AJR Am J Roentgenol. 2009;193:1124–7. 19770337 10.2214/AJR.09.2520

[6] Lee SY, Yang MS, Choi YH, Park CM, Park HW, Cho SH, et al. Stratified premedication strategy for the prevention of contrast media hypersensitivity in high-risk patients. Ann Allergy Asthma Immunol. 2017;118(339–344):e1. 10.1016/j.anai.2016.11.027 28087383

[7] Dillman JR, Ellis JH, Cohan RH, Strouse PJ, Jan SC. Frequency and severity of acute allergic-like reactions to gadolinium-containing i.v. contrast media in children and adults. AJR Am J Roentgenol. 2007;189:1533–8. 18029897 10.2214/AJR.07.2554

[8] Granata V, Cascella M, Fusco R, dell’Aprovitola N, Catalano O, Filice S, et al. Immediate adverse reactions to gadolinium-based MR contrast media: a retrospective analysis on 10,608 examinations. Biomed Res Int. 2016;2016:3918292. 27652261 10.1155/2016/3918292 PMC5019936

[9] Prince MR, Zhang H, Zou Z, Staron RB, Brill PW. Incidence of immediate gadolinium contrast media reactions. AJR Am J Roentgenol. 2011;196:W138–43. 21257854 10.2214/AJR.10.4885

[10] Aran S, Shaqdan KW, Abujudeh HH. Adverse allergic reactions to linear ionic gadolinium-based contrast agents: experience with 194, 400 injections. Clin Radiol. 2015;70:466–75. 25626627 10.1016/j.crad.2014.12.011

[11] Jung JW, Kang HR, Kim MH, Lee W, Min KU, Han MH, et al. Immediate hypersensitivity reaction to gadolinium-based MR contrast media. Radiology. 2012;264:414–22. 22550309 10.1148/radiol.12112025

[12] Fok JS, Smith WB. Hypersensitivity reactions to gadolinium-based contrast agents. Curr Opin Allergy Clin Immunol. 2017;17:241–6. 28537933 10.1097/ACI.0000000000000371

[13] Clement O, Dewachter P, Mouton-Faivre C, Nevoret C, Guilloux L, Bloch Morot E, et al. Immediate Hypersensitivity to Contrast Agents: the French 5-year CIRTACI Study. EClinicalMedicine. 2018;1:51–61. 31193689 10.1016/j.eclinm.2018.07.002 PMC6537532

[14] Gómez E, Ariza A, Blanca-López N, Torres MJ. Nonimmediate hypersensitivity reactions to iodinated contrast media. Curr Opin Allergy Clin Immunol. 2013;13:345–53. 23743515 10.1097/ACI.0b013e328362b926

[15] Nagai H, Nishigori C. A delayed reaction to the magnetic resonance imaging contrast agent gadobutrol. J Allergy Clin Immunol Pract. 2017;5:850–1. 28153713 10.1016/j.jaip.2017.01.001

[16] Ring J, Messmer K. Incidence and severity of anaphylactoid reactions to colloid volume substitutes. Lancet. 1977;1:466–9. 65572 10.1016/s0140-6736(77)91953-5

[17] Brockow K, Romano A, Aberer W, Bircher AJ, Barbaud A, Bonadonna P, et al. Skin testing in patients with hypersensitivity reactions to iodinated contrast media—a European multicenter study. Allery. 2009;64:234–41. 10.1111/j.1398-9995.2008.01832.x 19178403

[18] ESUR, Guidelines on Contrast Media. http://www.esur-cm.org, 10.0 edition, 2018.

[19] RosadoIngelmo A, DoñaDiaz I, CabañasMoreno R, MoyaQuesada MC, García-Avilés C, GarcíaNuñez I, et al. Clinical practice guidelines for diagnosis and management of hypersensitivity reactions to contrast media. J Investig Allergol Clin Immunol. 2016;26(3):144–55. 10.18176/jiaci.0058 27326981

[20] Costello JR, Kalb B, Martin DR. Incidence and risk factors for gadolinium-based contrast agent immediate reactions. Top Magn Reson Imaging. 2016;25:257–63. 27748714 10.1097/RMR.0000000000000109

[21] Kobayashi D, Takahashi O, Ueda T, Arioka H, Akaishi Y, Fukui T. Asthma severity is a risk factor for acute hypersensitivity reactions to contrast agents: a large-scale cohort study. Chest. 2012;141(5):1367–8. 22553277 10.1378/chest.11-3143

[22] Park HJ, Park JW, Yang MS, Kim MY, Kim SH, Jang GC, et al. Re-exposure to low osmolar iodinated contrast media in patients with prior moderate-to-severe hypersensitivity reactions: a multicentre retrospective cohort study. Eur Radiol. 2017;27:2886–93. 27975150 10.1007/s00330-016-4682-y

[23] Hsu Blatman KS, Sánchez-Borges M, Greenberger PA. Anaphylaxis in the radiology suite. J Allergy Clin Immunol Pract. 2020;8:1203–9. 32276689 10.1016/j.jaip.2020.01.014

[24] Tepetam FM, Çiftaslan N, Oruç Ö, Duman D, Ağca M, Bulut İ, et al. Should patients with risk factors be tested for hypersensitivity to contrast media: a prospective study. Radiol Med. 2016;121:660–6. 27117293 10.1007/s11547-016-0646-1

[25] Scherer K, Harr T, Bach S, Bircher AJ. The role of iodine in hypersensitivity reactions to radio contrast media. Clin Exp Allergy. 2010;40:468–75. 20210815 10.1111/j.1365-2222.2009.03361.x

[26] Sánchez-Borges M, Aberer W, Brockow K, Celik GE, Cernadas J, Greenberger PA, et al. Controversies in drug allergy: radiographic contrast media. J Allergy Clin Immunol Pract. 2019;7:61–5. 30573421 10.1016/j.jaip.2018.06.030

[27] Greenberger PA, Halwig JM, Patterson R, Wallemark CB. Emergency administration of radiocontrast media in high-risk patients. J Allergy Clin Immunol. 1986;77:630–4. 3958391 10.1016/0091-6749(86)90357-x

[28] Lasser EC, Berry CC, Talner LB, Santini LC, Lang EK, Gerber FH, et al. Pretreatment with corticosteroids to alleviate reactions to intravenous contrast material. N Engl J Med. 1987;317:845–9. 3627208 10.1056/NEJM198710013171401

[29] Freed KS, Leder RA, Alexander C, DeLong DM, Kliewer MA. Breakthrough adverse reactions to low-osmolar contrast media after steroid premedication. AJR Am J Roentgenol. 2001;176:1389–92. 11373198 10.2214/ajr.176.6.1761389

[30] Tramèr MR, von Elm E, Loubeyre P, Hauser C. Pharmacological prevention of serious anaphylactic reactions due to iodinated contrast media: systematic review. BMJ. 2006;333:675. 16880193 10.1136/bmj.38905.634132.AE PMC1584363

[31] Mervak BM, Davenport MS, Ellis JH, Cohan RH. Rates of breakthrough reactions in inpatients at high risk receiving premedication before contrast-enhanced CT. AJR Am J Roentgenol. 2015;205:77–84. 26102383 10.2214/AJR.14.13810

[32] Abe S, Fukuda H, Tobe K, Ibukuro K. Protective effect against repeat adverse reactions to iodinated contrast medium: premedication vs. changing the contrast medium. Eur Radiol. 2016;26:2148–54. 26427700 10.1007/s00330-015-4028-1

[33] Davenport MS, Cohan RH. The evidence for and against corticosteroid prophylaxis in at-risk patients. Radiol Clin North Am. 2017;55:413–21. 28126223 10.1016/j.rcl.2016.10.012

[34] American College of Radiology. ACR manual on contrast media version 10. Reston: American College of Radiology; 2015.

[35] Brunton L, Hilal-Dandan R, Knollman B. Goodman and Gilman’s the pharmacological basis of therapeutics. 13th ed. New York: McGraw Hill Higher Education; 2018.

[36] Waljee AK, Rogers MA, Lin P, Singal AG, Stein JD, Marks RM, et al. Short term use of oral corticosteroids and related harms among adults in the United States: population based cohort study. BMJ. 2017;357:j1415. 28404617 10.1136/bmj.j1415 PMC6284230

[37] Vernassiere C, Trechot P, Commun N, Schmutz JL, Barbaud A. Low negative predictive value of skin tests in investigating delayed reactions to radio-contrast media. Contact Dermatitis. 2004;50:359–66. 15274727 10.1111/j.0105-1873.2004.00367.x

[38] Seitz CS, Pfeuffer P, Raith P, Bröcker EB, Trautmann A. Radiocontrast media-associated exanthema: identification of cross-reactivity and tolerability by allergologic testing. Eur J Radiol. 2009;72:167–71. 18620831 10.1016/j.ejrad.2008.06.010

[39] Hasdenteufel F, Waton J, Cordebar V, Studer M, Collignon O, Luyasu S, et al. Delayed hypersensitivity reactions caused by iodixanol: an assessment of cross-reactivity in 22 patients. J Allergy Clin Immunol. 2011;128:1356–7. 21782229 10.1016/j.jaci.2011.05.034

[40] Torres MJ, Gomez F, Doña I, Rosado A, Mayorga C, Garcia I, et al. Diagnostic evaluation of patients with nonimmediate cutaneous hypersensitivity reactions to iodinated contrast media. Allergy. 2012;67:929–35. 22583135 10.1111/j.1398-9995.2012.02840.x

[41] Prieto-García A, Tomás M, Pineda R, Tornero P, Herrero T, Fuentes V, et al. Skin test-positive immediate hypersensitivity reaction to iodinated contrast media: the role of controlled challenge testing. J Investig Allergol Clin Immunol. 2013;23:183–9. 23967757

[42] Salas M, Gomez F, Fernandez TD, Doña I, Aranda A, Ariza A, et al. Diagnosis of immediate hypersensitivity reactions to radiocontrast media. Allergy. 2013;68:1203–6. 23991759 10.1111/all.12214

[43] Lerondeau B, Trechot P, Waton J, Poreaux C, Luc A, Schmutz JL, et al. Analysis of cross-reactivity among radiocontrast media in 97 hypersensitivity reactions. J Allergy Clin Immunol. 2016;137(633–635):e4. 10.1016/j.jaci.2015.07.035 26428955

[44] Morales-Cabeza C, Roa-Medellín D, Torrado I, De Barrio M, Fernández-Álvarez C, Montes-Aceñero JF, et al. Immediate reactions to iodinated contrast media. Ann Allergy Asthma Immunol. 2017;119:553–7. 29017901 10.1016/j.anai.2017.08.014

[45] Schrijvers R, Breynaert C, Ahmedali Y, Bourrain JL, Demoly P, Chiriac AM. Skin testing for suspected iodinated contrast media hypersensitivity. J Allergy Clin Immunol Pract. 2018;6:1246–54. 29371073 10.1016/j.jaip.2017.10.040

[46] Trautmann A, Brockow K, Behle V, Stoevesandt J. Radiocontrast media hypersensitivity: skin testing differentiates allergy from nonallergic reactions and identifies a safe alternative as proven by intravenous provocation. J Allergy Clin Immunol Pract. 2019;7:2218–24. 30980898 10.1016/j.jaip.2019.04.005

[47] Soria A, Masson N, Vial-Dupuy A, Gaouar H, Amsler E, Chollet-Martin S, et al. Allergological workup with half-dose challenge in iodinated contrast media hypersensitivity. Allergy. 2019;74:414–7. 30353926 10.1111/all.13638

[48] Kwon OY, Lee JH, Park SY, Seo B, Won H-K, Kang Y, et al. Novel strategy for the prevention of recurrent hypersensitivity reactions to radiocontrast media based on skin testing. J Allergy Clin Immunol Pract. 2019;7:2707–13. 31078762 10.1016/j.jaip.2019.04.036

[49] Kolenda C, Dubost R, Hacard F, Mullet C, Le Quang D, Garnier L, et al. Evaluation of basophil activation test in the management of immediate hypersensitivity reactions to gadolinium-based contrast agents: a five-year experience. J Allergy Clin Immunol Pract. 2017;5:846–9. 28341169 10.1016/j.jaip.2017.01.020

[50] Fernandez J, Blanca M, Moreno F, Garcia J, Segurado E, del Cano A, et al. Role of tryptase, eosinophil cationic protein and histamine in immediate allergic reactions to drugs. Int Arch Allergy Immunol. 1995;107:160–2. 7542066 10.1159/000236964

[51] Demoly P, Adkinson NF, Brockow K, Castells M, Chiriac AM, Greenberger PA, et al. International consensus on drug allergy. Allergy. 2014;69:420–37. 24697291 10.1111/all.12350

[52] Yoon SH, Lee SY, Kang HR, Kim J-Y, Hahn S, Park CM, et al. Skin tests in patients with hypersensitivity reaction to iodinated contrast media: a meta-analysis. Allergy. 2015;70:625–37. 25649510 10.1111/all.12589

[53] Brockow K, Garvey LH, Aberer W, Atanaskovic-Markovic M, Barbaud A, Bilo MB, et al. Skin test concentrations for systemically administered drugs—an ENDA/EAACI Drug Allergy Interest Group position paper. Allergy. 2013;68(6):702–12. 23617635 10.1111/all.12142

[54] Sesé L, Gaouar H, Autegarden JE, Alari A, Amsler E, Vial-Dupuy A, et al. Immediate hypersensitivity to iodinated contrast media: diagnostic accuracy of skin tests and intravenous provocation test with low dose. Clin Exp Allergy. 2016;46:472–8. 26750091 10.1111/cea.12703

[55] Chiriac AM, Audurier Y, Bousquet PJ, Demoly P. Clinical value of negative skin tests to gadolinium contrast agents. Allergy. 2011;66:1504–6. 21854399 10.1111/j.1398-9995.2011.02690.x

[56] Kim SH, Jo EJ, Kim MY, Lee SE, Kim MH, Yang MS, et al. Clinical value of radiocontrast media skin tests as a prescreening and diagnostic tool in hypersensitivity reactions. Ann Allergy Asthma Immunol. 2013;110:258–62. 23535089 10.1016/j.anai.2013.01.004

[57] Lee JH, Kwon OY, Park SY, Seo B, Won HK, Kang Y, et al. Validation of the prescreening intradermal skin test for predicting hypersensitivity to iodinated contrast media: a prospective study with ICM challenge. J Allergy Clin Immunol Pract. 2020;8:267–72. 31408712 10.1016/j.jaip.2019.08.001

