# Correction to: SIRM‑SIAAIC consensus, an Italian document on management of patients at risk of hypersensitivity reactions to contrast media

**DOI:** 10.1186/s12948-024-00190-1

**Published:** 2024-08-22

**Authors:** Maria Teresa Costantino, Laura Romanini, Francesco Gaeta, Fulvio Stacul, Rocco Luigi Valluzzi, Matteo Passamonti, Patrizia Bonadonna, Giovanni Cerri, Stefano Pucci, Paolo Ricci, Eleonora Savi, Michele Galluzzo, Marina Mauro, Emanuele Grassedonio, Mona Rita Yacoub, Alfonso Reginelli, Sergio Testi, Erminia Ridolo, Eustacchio Nettis, Elisabetta Di Leo, Oliviero Rossi, Paolo Montuschi, Cristoforo Incorvaia, Antonino Romano

**Affiliations:** 1 Allergy Unit, Department of Medicine, ASST Mantova, Mantua, Italy; 2 Radiology, Istituti Ospitalieri di Cremona, ASST Cremona, Cremona, Italy; 3 Allergy Unit, Columbus Hospital, Fondazione Policlinico Universitario Agostino Gemelli IRCCS, Via Moscati n.30, Rome, Italy; 4 Department of Radiology, Ospedale Maggiore, Azienda Sanitaria Universitaria Integrata di Trieste, Trieste, Italy; 5 Allergy Unit, Bambino Gesù Children’s Hospital, IRCCS, Rome, Vatican City, Italy; 6 Department of Radiology-ASST di Lodi, Lodi, Italy; 7 Allergy Unit, Azienda Ospedaliera Universitaria Integrata of Verona, Verona, Italy; 8 Department of Radiology, ASST Spedali Civili di Brescia, Brescia, Italy; 9 Allergy Unit. General Hospital, Civitanova Marche, Milan, Italy; 10 Department of Radiology, Oncologiche ad Anatomopatologiche, Azienda Policlinico Umberto I, Sapienza Università di Roma, Rome, Italy; 11 Departmental Unit of Allergology, Guglielmo da Saliceto Hospital, Piacenza, Italy; 12 Department of Radiology, Azienda Ospedaliera San Camillo Forlanini, Ospedale San Camillo, Rome, Italy; 13 Allergy Unit, S. Anna Hospital, Como, Italy; 14 Department of Radiology, Dipartimento di Biopatologia e Biotecnologie Mediche, Policlinico Paolo Giaccone, Università degli Studi di Palermo, Palermo, Italy; 15 Allergy and Immunology Department, IRCCS San Raffaele Hospital, Milan, Italy; 16 Department of Radiology & Radiotherapy, University of Campania ‘Luigi Vanvitelli’, Naples, Italy; 17 Allergy and Clinical Immunology Unit, San Giovanni di Dio’s Hospital, Florence, Italy; 18 Department of Medicine and Surgery Clinical, University of Parma, Via Gramsci 14, 43126 Parma, Italy; 19 Department of Emergency and Organ Transplantation, School of Allergology and Clinical Immunology, University of Bari Aldo Moro, Bari, Italy; 20 Section of Allergy and Clinical Immunology, Unit of Internal Medicine, “F. Miulli” Hospital, Acquaviva delle Fonti, Bari, Italy; 21 Allergy Unit, Azienda Ospedaliero Universitaria Careggi, Florence, Italy; 22 Pharmacology Unit, Fondazione Policlinico Universitario Agostino Gemelli IRCCS, Rome, Italy; 23 Department of Pharmacology, Faculty of Medicine, Catholic University of the Sacred Heart, Rome, Italy; 24 Cardiac/Pulmonary Rehabilitation, ASST Pini-CTO, Milan, Italy; 25 IRCCS Oasi Maria S.S., Troina & Fondazione Mediterranea G.B. Morgagni, Catania, Italy

**Correction to: Clinical and Molecular Allergy (2020) 18:13**

10.1186/s12948-020-00128-3

Following publication of the original article [1], the licence information was missing from this article and should have been ‘This article is licensed under a Creative Commons attribution 4.0 international license, which permits use, sharing, adaptation, distribution and reproduction in any medium or format, as long as you give appropriate credit to the original author(s) and the source, provide a link to the Creative Commons licence, and indicate if changes were made. The images or other third party material in this article are included in the article’s Creative Commons licence, unless indicated otherwise in a credit line to the material. If material is not included in the article’s Creative Commons licence and your intended use is not permitted by statutory regulation or exceeds the permitted use, you will need to obtain permission directly from the copyright holder. To view a copy of this licence, visit http://creativecommons.org/licenses/by/4.0/. The Creative Commons Public Domain Dedication waiver (http://creativecommons.org/publicdomain/zero/1.0/) applies to the data made available in this article, unless otherwise stated in a credit line to the data’

The original article has been corrected.
